# Molecular mechanism of the common and opposing cosolvent effects of fluorinated alcohol and urea on a coiled coil protein

**DOI:** 10.1002/pro.4763

**Published:** 2023-10-01

**Authors:** Noa Nakata, Ryuichi Okamoto, Tomonari Sumi, Kenichiro Koga, Takeshi Morita, Hiroshi Imamura

**Affiliations:** ^1^ Department of Chemistry, Faculty of Science Okayama University Okayama Japan; ^2^ Graduate School of Information Science, University of Hyogo Kobe Hyogo Japan; ^3^ Research Institute for Interdisciplinary Science, Okayama University Okayama Japan; ^4^ Department of Chemistry Graduate School of Science, Chiba University Chiba Japan; ^5^ Department of Bio‐Science Nagahama Institute of Bio‐Science and Technology Nagahama Shiga Japan

**Keywords:** 2,2,2‐trifluoroethanol, cosolvent effects, preferential binding parameter, protein folding stability, urea

## Abstract

Alcohols and urea are widely used as effective protein denaturants. Among monohydric alcohols, 2,2,2‐trifluoroethanol (TFE) has large cosolvent effects as a helix stabilizer in proteins. In contrast, urea efficiently denatures ordered native structures, including helices, into coils. These opposing cosolvent effects of TFE and urea are well known, even though both preferentially bind to proteins; however, the underlying molecular mechanism remains controversial. Cosolvent‐dependent relative stability between native and denatured states is rigorously related to the difference in preferential binding parameters (PBPs) between these states. In this study, GCN4‐p1 with two‐stranded coiled coil helices was employed as a model protein, and molecular dynamics simulations for the helix dimer and isolated coil were conducted in aqueous solutions with 2 M TFE and urea. As 2 M cosolvent aqueous solutions did not exhibit clustering of cosolvent molecules, we were able to directly investigate the molecular origin of the excess PBP without considering the enhancement effect of PBPs arising from the concentration fluctuations. The calculated excess PBPs of TFE for the helices and those of urea for the coils were consistent with experimentally observed stabilization of helix by TFE and that of coil by urea. The former was caused by electrostatic interactions between TFE and side chains of the helices, while the latter was attributed to both electrostatic and dispersion interactions between urea and the main chains. Unexpectedly, reverse‐micelle‐like orientations of TFE molecules strengthened the electrostatic interactions between TFE and the side chains, resulting in strengthening of TFE solvation.

## 1 INTRODUCTION

Urea and alcohols are both routinely used as protein denaturing agents in in vitro unfolding and refolding experiments (Kauzmann, 1959; Mirsky and Pauling, 1936; Tanford, 1970); however, their mechanisms of action are different. Urea destabilizes native ordered structure of proteins, including helical secondary structures, and induces disordered, coiled structures, whereas the addition of alcohol destabilizes the native structure of proteins, resulting in helical structures. To understand the molecular mechanisms underlying the two opposing cosolvent effects of urea and alcohols as well as to gain insights into chemical denaturation of proteins, the free energies for transferring amino acid side chains and those for transferring peptide backbone from water into the aqueous mixtures of these cosolvents have been measured experimentally (Nozaki and Tanford, 1963, 1971; Tanford, 1970; Watlaufer et al., 1964; Whitney and Tanford, 1962). It has been found that the solubility of most hydrocarbons and protein side chain analogues increases upon their transfer from water to aqueous solutions of urea and alcohols (Nozaki and Tanford, 1963, 1971; Tanford, 1970; Watlaufer et al., 1964; Whitney and Tanford, 1962). These experiments suggest that the exposure of hydrophobic side chains into those aqueous solutions upon unfolding of proteins is favorable, and leads to protein denaturation. This scenario seems to be energetically consistent with urea‐induced coil formation (e.g., unfolded structure) and alcohol‐induced formation of a helix‐rich conformation (e.g., a molten globule structure) because hydrophobic side chains of both the coils of unfolded proteins and the helices in molten globules are more exposed to the solutions than those in the native structures of proteins. In addition, it has been experimentally demonstrated that the transfer of a peptide backbone from water to aqueous urea solution increases its solubility, while its transfer to aqueous alcohol solutions decreases its solubility (Nozaki and Tanford, 1963, 1971; Tanford, 1970). Therefore, considering the fact that the exposure of the peptide backbone of a helix to the solvent is lower than that of a coil, it is found that the free energies of transfer of peptide backbone from water to aqueous mixtures of these cosolvents are energetically consistent with the coil induction by urea and helix induction by alcohols.

According to the classical formalism by Tanford (1970), the free energy of chemical denaturation can be estimated by utilizing the free energy of transfer of each constituent group. However, such formalism based on the exposed area of each group to the solvent, that is, solvent accessible surface area (SASA) of each group, assumes the group additivity of the solvation free energy; however, it does not consider the cavity formation free energy for buried residues inside the protein, which is a major drawback as mentioned in our previous study (Sumi and Imamura, 2021). Thus, a theoretical study of the cosolvent effects based on a complete treatment of folded and unfolded structures of protein without assuming the group additivity in the SASA‐based formalism is desirable to gain insights into the molecular mechanisms underlying the opposing cosolvent effects induced by urea and alcohols.

In general, the ability of urea as a protein denaturant has been explained based on two different molecular mechanisms: the direct and indirect interaction mechanisms. In the indirect mechanism, urea disrupts the network structures of water, thereby weakening the hydrophobic interactions, or promotes the hydration of hydrophobic groups (Bennion and Daggett, 2003; Chen et al., 2007; Frank and Franks, 1967; Hammes and Schimmel, 1967). Furthermore, urea strengthens the hydrogen bonds between water and protein such that water strongly solvates the protein (Caballero‐Herrera et al., 2005; Caflisch and Karplus, 1999), resulting in the denaturation of the protein. On the other hand, in the direct mechanism, urea unfolds proteins via direct electrostatic and dispersion interactions with the proteins (Auton et al., 2007; Berteotti et al., 2011; Canchi et al., 2010; Das and Mukhopadhyay, 2009; Hua et al., 2008; Lim et al., 2009; Makhatadze and Privalov, 1992; Robinson and Jencks, 1965; Yang et al., 2012). Among these studies, compromise views that both direct and indirect mechanisms are important in urea‐induced denaturation are included (Bennion and Daggett, 2003; Caballero‐Herrera et al., 2005; Caflisch and Karplus, 1999; Das and Mukhopadhyay, 2009).

The cosolvent effects of alcohols on proteins and peptides have been studied extensively for more than the last five decades (Herskovits et al., 1970; Tanford, 1968). It has been concluded that the effectiveness of the alcohols as protein denaturants increases with increasing chain length or hydrocarbon content of the alcohol molecule (Herskovits et al., 1970). 2,2,2‐Trifluoroethanol (TFE) has often been used to stabilize the α‐helical structure in denatured proteins and their fragments because of its marked ability of helix induction (Hamada et al., 1995; Jasanoff and Fersht, 1994; Shiraki et al., 1995; Sonnichsen et al., 1992; Yang et al., 1995). However, why TFE possesses such high ability of helix induction among alcohols remains unclear. The effects of alcohols as denaturants can be partly attributable to the polarity of alcohols being lower than that of water (Tanford, 1968). Alcohols with a low polarity are considered to strengthen intramolecular electrostatic interactions, such as hydrogen bonds, within proteins, resulting in the stabilization of helical structures. Notably, a high correlation between the relative dielectric constant of organic solvents, including alcohols, and extent of conformational transition of a protein has been previously shown (Uversky et al., 1997). The free energy of transfer of nonpolar side chains from water to an aqueous solution of alcohol, for example, ethanol, is negative, while that of peptide backbone is positive. The observations may stem from the low polarity of alcohols (Liu and Bolen, 1995; Tanford, 1970). Therefore, both the exposure of nonpolar side chains to the aqueous alcohol solution and burial of peptide backbone inside the protein structure are favorable for the formation of α‐helix. However, the potency of fluorine‐substituted alcohols, including TFE and 1,1,1,3,3,3‐hexafluoro‐2‐propanol (HFIP), to transform the structure of proteins is much higher than that predicted based on the low polarity effects of alcohols. Thus, it has been pointed out that other unknown factors contribute to the cosolvent effects induced by these alcohols (Hong et al., 1999). When considering the high ability of helix induction by TFE and HFIP, aggregation or clustering of these alcohol molecules is thought to play an important role such that the clusters of bulky alcohol molecules provide a highly hydrophobic micelle‐like environment, where local polarity decreases and hydrogen bonds within protein are strengthened (Hong et al., 1999). However, it has recently been shown that TFE‐induced conformational transition of a protein was already significant at concentrations much lower than the concentration of clustering of TFE molecules, which was anomalous for TFE among alcohols (Ohgi et al., 2021). Therefore, apart from the clustering effects (hereafter, also denoted as concentration fluctuation), other factors that affect protein conformation even at low alcohol concentrations, where concentration fluctuations are very small, must play an important role in the cosolvent effects of these fluorine‐substituted alcohols. However, previous studies have not considered the differences between high and low concentration regimes of alcohols when discussing alcohol‐induced helix formation.

In general, cosolvent‐dependent relative stability between native and denatured states is rigorously related to the difference in preferential binding parameters (PBPs) of a cosolvent between these two states (Pierce et al., 2008; Smith, 2006). However, few molecular dynamics (MD) studies have discussed the difference in the PBPs between folded and unfolded states (Canchi and García, 2011; Su and Dias, 2017; Vymětal et al., 2016), although many have calculated the PBPs for either one of these two conformational states or a mixed state at given cosolvent concentrations (Canchi and García, 2011; de Oliveira and Martínez, 2019; Ganguly et al., 2018; Gerig, 2019; Pereira et al., 2022; Su and Dias, 2017; Vymětal et al., 2016). The molecular mechanisms underlying protein denaturation by urea and TFE are still controversial because such PBP analyses are not sufficient to gain insights into the influence of cosolvents on the stabilization of each conformational state of the protein. In this study, we have focused on the difference in the PBPs between folded and unfolded states and aimed to reveal the molecular origin of the opposing cosolvent effects induced by urea and TFE by identifying the dominant factors that yield the difference in the PBPs. The calculated excess PBPs of TFE for the helices than the coils and those of urea for the coils than the helices were consistent with experimentally observed stabilization of helix by TFE and that of coil by urea. The former was caused by electrostatic interactions between TFE and side chains of the helices, while the latter was attributed to both electrostatic and dispersion interactions between urea and the main chains. The molecular origin of the opposing cosolvent effects can be attributed to the chemical nature of these cosolvent molecules in that the OH groups of TFE molecule prefer to interact with polar side chains, whereas urea molecules prefer to interact with peptide backbones.

## 2 BACKGROUND OF THEORETICAL ANALYSIS

### 2.1 Preferential binding and effects of cosolvents on protein stability

As mentioned above, the PBP of cosolvent over water for protein, $\Gamma_{23}$, is related to cosolvent‐dependent structural stability of proteins (Pierce et al., 2008; Smith, 2006). Here, the primary solvent (water), biomolecular solute (protein), and cosolvent species are referred to as components 1, 2, and 3, respectively. In the present study, we employed the two‐stranded coiled‐coil domain of the yeast transcription activator GCN4‐p1 as the model protein (component 2) (O'Shea et al., 1989, 1991). The two‐stranded helices are stabilized by direct interactions between the nonpolar residues repeatedly appearing on the helix dimer interface (Sumi and Imamura, 2021). This protein is crystallizable, and thus, the native structure has been defined exactly (O'Shea et al., 1989, 1991); this is superior to a poorly defined “native” structure of other helical models, such as an alanine‐based peptide. TFE can stabilize the α‐helices of GCN4‐p1 (Kentsis and Sosnick, 1998). The high helical propensity of GCN4‐p1 allows the observation of the stabilization of α‐helices upon addition of TFE as previous studies have suggested that TFE efficiently induces α‐helix formation in peptides and proteins with high intrinsic helical propensities (Hamada et al., 1995; Shiraki et al., 1995; Yang et al., 1995). In addition, the monomer helix isolated from GCN4‐p1 can be coiled in an aqueous solution and duly sampled by MD simulation. These characteristics offer an advantage in examining the opposing cosolvent effects, namely, the stabilization of helices by TFE and that of coils by urea. While high concentration of TFE induces dissociation of the dimer of coiled coils probably including GCN4‐p1, it is expected that this effect of TEF is negligible or much smaller when the concentration is low, for example, < ~15% (v/v), as used in the present study (Corrêa and Farah, 2007).

PBP for the two‐stranded helices of GCN4‐p1, $\Gamma_{23}^{h}$, and that for two isolated coils, $\Gamma_{23}^{c}$, can be given by Kirkwood–Buff (KB) integrals $G_{\mathrm{ij}}^{\alpha }$ as (Ohgi et al., 2021; Pierce et al., 2008)

(1) $$\Gamma_{23}^{\alpha }=\rho_{3}\left(G_{23}^{\alpha }-G_{21}^{\alpha }\right),\left(\alpha =h\;\text{or}\;c\right),$$

where $\rho_{3}$ is the number density of the cosolvent. The KB integrals are defined by

(2) $$G_{\mathrm{ij}}=\int_{0}^{\infty }\left[g_{\mathrm{ij}}\left(r\right)-1\right]4\pi r^{2}\mathrm{dr},$$

where $g_{\mathrm{ij}}\left(r\right)$ is a radial distribution function between components *i* and *j*. The difference between these PBPs is related to the difference between the standard chemical potentials of the helix dimer (${\bar{\mu }}_{\text{helix}}$) and two isolated coils (${\bar{\mu }}_{\text{coil}}$), and the Gibbs energy of the helix dimer and that of coils in the infinite dilution of the protein, $\Delta G_{\text{helix}}$, as follows (Ohgi et al., 2021; Pierce et al., 2008):

(3a) $$\begin{array}{rcl} \Delta \Gamma_{23} & \equiv & \Gamma_{23}^{h}-\Gamma_{23}^{c}=-\frac{\beta }{a_{33}}{\left(\frac{\partial \Delta G_{\text{helix}}}{\partial \ln\rho_{3}}\right)}_{T,P}\\ & = & -\frac{\beta \rho_{3}}{a_{33}}{\left(\frac{\partial \Delta G_{\text{helix}}}{\partial \rho_{3}}\right)}_{T,P}, \end{array}$$

(3b) $$\Delta G_{\text{helix}}\equiv {\bar{\mu }}_{\text{helix}}-{\bar{\mu }}_{\text{coil}}.$$

Here, $\beta =1/N_{A}k_{B}T$ ($N_{A}$ is the Avogadro constant, $k_{B}$ is the Boltzmann constant, and T is the temperature) and $a_{33}$ is provided using the bulk KB integrals as follows (Ohgi et al., 2021; Pierce et al., 2008):

(4) $$a_{33}=\frac{1}{1+\rho_{3}\left(G_{33}-G_{31}\right)}.$$

If the helix dimer is more efficiently stabilized by an addition of cosolvent than the two isolated coils, $\Delta G_{\text{helix}}$ becomes negative and, $\Delta \Gamma_{23}$ should be positive when $a_{33}$ is positive, indicating that the cosolvent more strongly solvate the helix dimer (also see Appendix S1).

Empirically, $\Delta G_{\text{helix}}$ can be established as a linear function of cosolvent concentration [Co‐sol] in units of mol/L or M as follows:

(5) $$\Delta G_{\text{helix}}=\Delta G_{0}-m\left[\mathrm{Co}-\mathrm{sol}\right],$$

where $\Delta G_{0}$ is $\Delta G_{\text{helix}}$ at $\left[\mathrm{Co}-\mathrm{sol}\right]=0$ and m is the slope of $\Delta G_{\text{helix}}$ with respect to $\left[\mathrm{Co}-\mathrm{sol}\right]$, which is called m‐value (Hirota et al., 1997, 1998; Ibarra‐Molero et al., 2004; Matousek et al., 2007). If the aqueous mixture of cosolvent has large concentration fluctuations, $a_{33}$ becomes smaller than 1, thereby, $\Delta \Gamma_{23}$ is amplified by $a_{33}$, whereas if the mixture is homogeneous and has neither large concentration fluctuations nor clustering of cosolvent molecules, $a_{33}$ is nearly equal to 1, resulting in no amplification of $\Delta \Gamma_{23}$ by $a_{33}$ (see Equation 3a). If Equation (5) is substituted into Equation (3a), the following is obtained (Ohgi et al., 2021; Pierce et al., 2008)

(6) $$m=\Delta \Gamma_{23}\frac{a_{33}}{\beta \rho_{3}},$$

Thus, if $\Delta \Gamma_{23}$ is determined by performing MD simulations at a certain cosolvent concentration $\rho_{3}$ (number density), the m‐value can be obtained from Equation (6).

In our previous study, KB integrals for aqueous TFE bulk solutions were determined by performing small‐angle X‐ray scattering measurements (Ohgi et al., 2021). Utilizing those KB integrals, $a_{33}$ was calculated from Equation (4) as a function of [TFE]. We found that, $a_{33}$ was nearly equal to 1 at concentrations less than ~2 M, that is, mole fraction of TFE being approximately 0.04 (Ohgi et al., 2021). Thus, the concentration dependence of $\Delta G_{\text{helix}}$ depends only on $\Delta \Gamma_{23}$ at the low concentrations of TFE (see Equations 5 and 6). This implies that the mechanism of TFE‐mediated helix stabilization can potentially be revealed by investigating why the PBP of TFE to helices is larger than that to coils, namely, the molecular origin of $\Delta \Gamma_{23}>0$. Meanwhile, interestingly, $a_{33}$ was calculated from Equation (4) utilizing experimentally determined KB integrals for aqueous urea bulk solutions to be nearly equal to 1 over the wide range of [urea] up to 8 M (Chitra and Smith, 2002), implying that the concentration dependence of $\Delta G_{\text{helix}}$ is governed only by $\Delta \Gamma_{23}$. Thus, in the same manner, the mechanism underlying coil formation by urea should be reduced to the reason why the PBP of urea for coils is larger than that for helices, namely, the molecular origin on $\Delta \Gamma_{23}<0$. Based on this thermodynamic analysis, in this study, we investigated the molecular mechanism yielding positive and negative values of $\Delta \Gamma_{23}$ in the aqueous TFE and urea solutions, respectively, at the same molar fraction (viz. 0.04) chosen for comparison.

## 3 RESULTS AND DISCUSSION

### 3.1 Excess preferential solvation of cosolvent $\Delta \Gamma_{23}$

**FIGURE 1:**
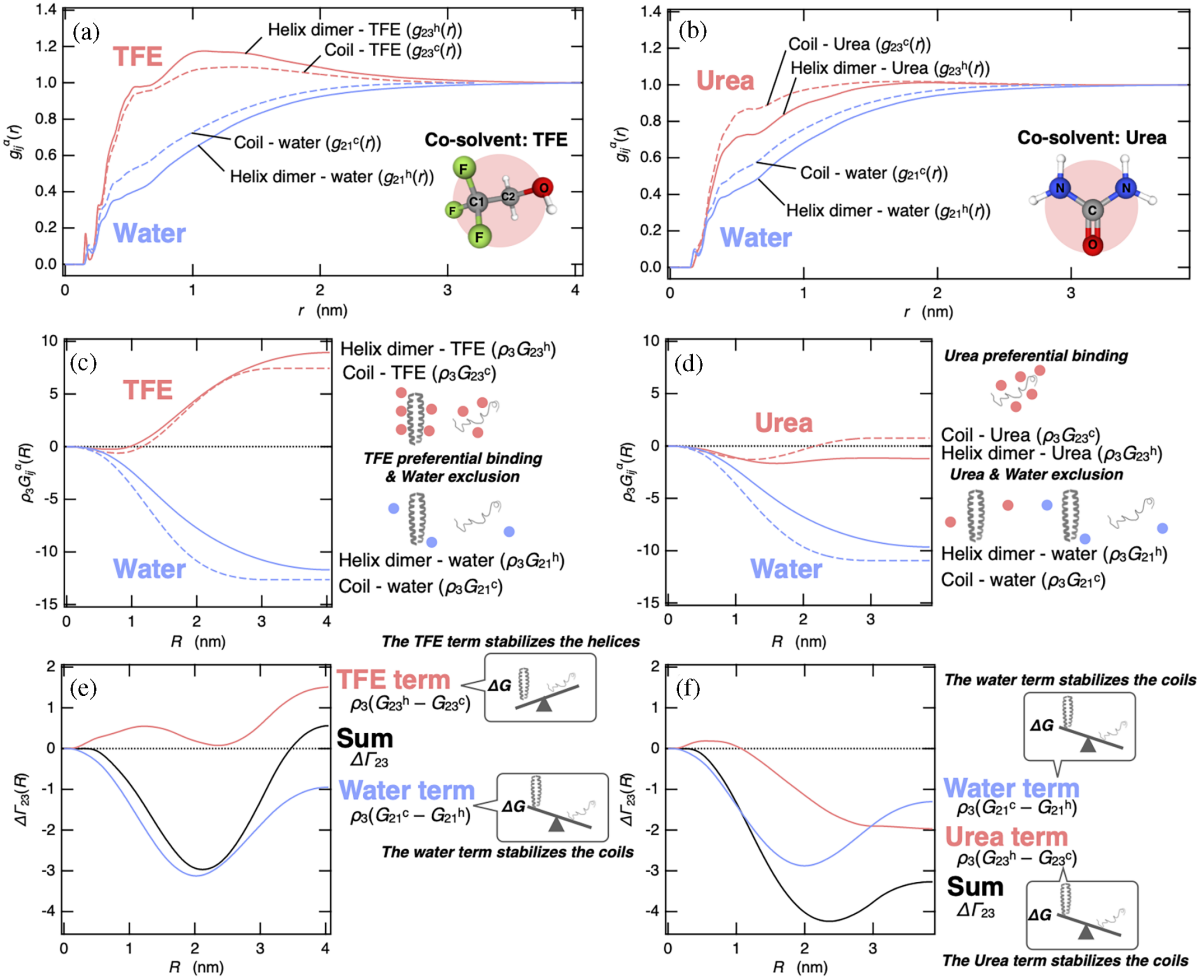
The excess preferential solvation of TFE and urea for the helix dimer, $∆\Gamma_{23}$, is obtained as a positive and negative value, respectively, indicating the stabilization of the helix dimer by TFE and that of the coils by urea. (a and b) The radial distribution functions between all the atoms of the protein and all the atoms of the cosolvent $g_{23}^{\alpha }\left(r\right)$ and between all the atoms of the protein and all the atoms of water $g_{21}^{\alpha }\left(r\right)$ in the aqueous TFE (a) and urea (b) solution, where the superscript α is helix dimer or coil monomer. (c and d) The KB integrals multiplied by the number density of cosolvent, $\rho_{3}G_{\mathrm{ij}}^{\alpha }\left(R\right)$, in the aqueous TFE (c) and urea (d) solutions are shown as a function of the upper limit in the KB integral, R, defined by Equation (7a). (e and f) The excess preferential solvation of the cosolvent toward the helix dimer, $∆\Gamma_{23}\left(R\right)$, cosolvent term, and water term as defined by Equation (8) in the aqueous TFE (e) and urea (f) solutions are shown as a function of the upper limit in the KB integral. KB, Kirkwood–Buff; TFE, 2,2,2‐trifluoroethanol.

The KB integrals $G_{23}^{\alpha }$ and $G_{21}^{\alpha }$ for the helix dimer (α=h) and coil monomer (α=c) that are needed to determine $\Delta \Gamma_{23}$ (see Equation 1) were calculated from the integrals of the radial distribution functions between protein and cosolvent $g_{23}^{\alpha }\left(r\right)$ and between protein and water $g_{21}^{\alpha }\left(r\right)$ for the conformations of the helix dimer and coil:

(7a) $$G_{\mathrm{ij}}^{\alpha }\left(R\right)\equiv \left\{\begin{array}{c} \int_{0}^{R}\left[g_{\mathrm{ij}}^{h}\left(r\right)-1\right]4\pi r^{2}\mathrm{dr}\\ 2\int_{0}^{R}\left[g_{\mathrm{ij}}^{c}\left(r\right)-1\right]4\pi r^{2}\mathrm{dr} \end{array}\right.\;\begin{array}{c} \left(\alpha =h\right)\\ \left(\alpha =c\right) \end{array},$$

(7b) $$G_{\mathrm{ij}}^{\alpha }=\lim_{R\to \infty }G_{\mathrm{ij}}^{\alpha }\left(R\right),$$

where R is the upper limit of the integral in Equation (7a), and Equation (7b) indicates that R should be sufficiently large such that $G_{\mathrm{ij}}^{\alpha }\left(R\right)$ becomes asymptotically constant. In Equation (7a), $g_{\mathrm{ij}}^{c}\left(r\right)$ is the radial distribution function for the coil monomer, and thus, the factor 2 appears to yield the KB integral for two isolated coil monomers. $g_{23}^{\alpha }\left(r\right)$ and $g_{21}^{\alpha }\left(r\right)$ that were used to determine the KB integrals are displayed in Figure 1a,b, for aqueous TFE and urea solution, respectively. Here, $g_{23}^{\alpha }\left(r\right)$ and $g_{21}^{\alpha }\left(r\right)$ were calculated as the average of radial distribution functions between all the atoms of the protein and all the atoms of solvent molecules, namely, the cosolvent molecule (j=3) and water molecule (j=1), respectively. TFE molecules were much more strongly attracted to the helix dimer than the coil monomer (Figure 1a), while urea molecules marginally preferred the coil monomer over the helix dimer (Figure 1b). By contrast, in both the solutions, water molecules were more largely excluded from the near protein molecule by the helix dimer than by the coil monomer, that is, $g_{21}^{h}\left(r\right)$ was less than $g_{21}^{c}\left(r\right)$ near the protein (at small *r*), simply because of the molecular size of the helix dimer being larger than that of the coil (Figure 1a,b).

In Figure 1c,d, the KB integrals as a function of *R*, ${\rho_{3}G}_{23}^{\alpha }\left(R\right)$ and ${\rho_{3}G}_{21}^{\alpha }\left(R\right)$ in the aqueous TFE and urea solution, respectively, are shown as a function of the upper limit of the integral R on Equation (7a). From these results and Equation (1), it was found that both TFE and urea accumulated preferentially around the protein, that is, $\Gamma_{23}^{\alpha }>0$, regardless of the conformations, as shown in the previous studies (Canchi and García, 2011; Vymětal et al., 2016). While $g_{21}^{c}\left(r\right)$ was larger than $g_{21}^{h}\left(r\right)$ in both the aqueous solutions (Figure 1a,b), $G_{21}^{c}$ was negatively larger than $G_{21}^{h}$ in both the aqueous solutions (Figure 1c,d) due to the factor 2 on the coils as represented in Equation (7a). This fact is not trivial and the reasons will be discussed later. In the TFE solution, as expected from the larger maximum in $g_{23}^{h}\left(r\right)$ (Figure 1a), ${\rho_{3}G}_{23}^{h}$ for the helix dimer had a large positive value and was larger than ${\rho_{3}G}_{23}^{c}$ for the coils (Figure 1c). By contrast, when urea was used as a cosolvent, as expected from the small maximum in $g_{23}^{\alpha }\left(r\right)$ for both the conformations (Figure 1b), ${\rho_{3}G}_{23}^{\alpha }$ had a small positive and small negative value for the coils and helices, respectively, indicating that direct binding of urea molecules to the protein may not be as strong as generally assumed. While the magnitudes of ${\rho_{3}G}_{23}^{\alpha }$ for the two conformations were relatively small, we observed that there was a significant difference between ${\rho_{3}G}_{23}^{c}$ and ${\rho_{3}G}_{23}^{h}$ as shown in Figure 1d.

The excess preferential solvation of cosolvent $\Delta \Gamma_{23}$ was obtained from these KB integrals as follows:

(8) $$\Delta \Gamma_{23}=\rho_{3}\left(G_{23}^{h}-G_{23}^{c}\right)+\rho_{3}\left(G_{21}^{c}-G_{21}^{h}\right),$$

where the first and second terms on the right‐hand side are the cosolvent and water term, respectively, and $\Delta \Gamma_{23}$ is given as the sum of them. $\Delta \Gamma_{23}$, the cosolvent term, and the water term provided by the KB integrals, are shown as a function of the upper limit of the integral, R, for the aqueous TFE (Figure 1e) and urea (Figure 1f) solutions. In both the aqueous mixtures of cosolvents, the water term $\rho_{3}\left(G_{21}^{c}-G_{21}^{h}\right)$ decreased $\Delta \Gamma_{23}$ because of $G_{21}^{c}<G_{21}^{h}$ as seen in Figure 1c,d, indicating that the water term stabilized the coils. Notably, the water and cosolvent terms became zero without the presence of cosolvent because of $\rho_{3}$ in these terms. Even though the water term is unfavorable for the helix induction, the cosolvent term $\rho_{3}\left(G_{23}^{h}-G_{23}^{c}\right)$ was sufficiently large due to the stronger solvation of TFE for the helix dimer than that for the coils ($G_{23}^{h}>G_{23}^{c}$), such that $\Delta \Gamma_{23}$ became positive and the helix dimer was stabilized by the cosolvent effect by TFE (Figure 1e). On the other hand, in the urea solution, in addition to the water term, the cosolvent term was favorable for the coil formation (Figure 1f). Thus the large stabilization of the coils was induced owing to the cosolvent effect by urea, although the solvation of urea toward the coils was not as strong, as shown in Figure 1d. Notably, the effect of water exclusion by the coils, which relatively stabilizes the coils, should be regarded as one of cosolvent effects that is usually not accounted for during protein denaturation by urea.

**FIGURE 2:**
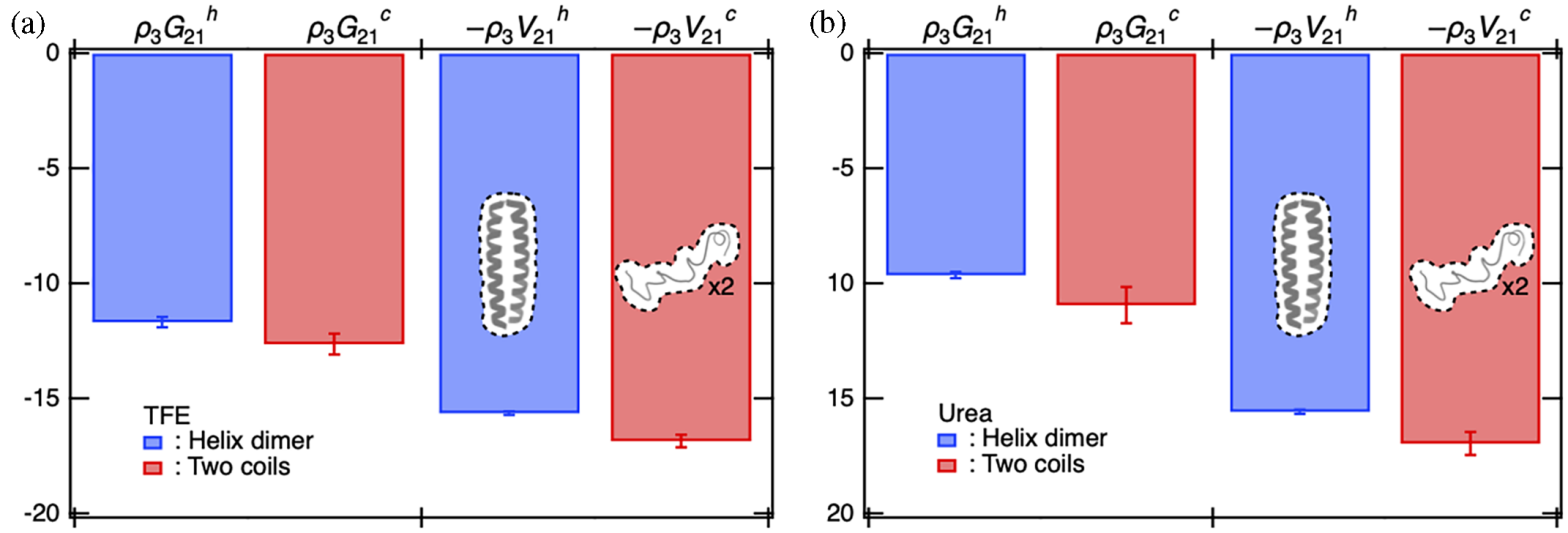
The water term with respect to $∆\Gamma_{23}$, namely, $\rho_{3}\left(G_{21}^{c}-G_{21}^{h}\right)$, is mainly attributable to the difference between the helix dimer and coils in the excluded volume of the protein, $-\rho_{3}\left(V_{21}^{c}-V_{21}^{h}\right)$. (a) The aqueous TFE solution. (b) The aqueous urea solution. Illustration indicates the excluded volume of the protein (the volume inside the dashed line). TFE, 2,2,2‐trifluoroethanol.

Herein, we delineated the common molecular origin of the negative value in the water term $\rho_{3}\left(G_{21}^{c}-G_{21}^{h}\right)$ stabilizing coil for TFE and urea. This term plays an important role especially for urea‐mediated denaturation. We observed $G_{21}^{c}<G_{21}^{h}$ for both the aqueous mixtures of cosolvents, implying that it does not depend on the chemical nature of the cosolvent molecules. Because the protein's partial specific volume ($v_{2}$) largely contributes to $G_{21}^{\alpha }$ as mentioned as $G_{21}^{\alpha }=k_{B}T\kappa_{T}-v_{2}$ in the infinite dilution of cosolvent (Shibuta and Imamura, 2018), where $\kappa_{T}$ is the isothermal compressibility of water, we determined the excluded volume of the protein $V_{21}^{\alpha }$ for the helix dimer (α=h) and coils (α=c) by using 0.14 nm as the radius of a water molecule, and compared ${-V}_{21}^{\alpha }$ with the KB integrals $G_{21}^{\alpha }$ (Figure 2). In both the aqueous mixtures of cosolvents, $-V_{21}^{c}$ was negatively larger than ${-V}_{21}^{h}$ such that the coils had more voids that are impenetrable for water molecules than the helix dimer (see Figure 2). The absolute value of $G_{21}^{\alpha }$ for both the conformations was smaller than that of $V_{21}^{\alpha }$ as the excluded volume effect by the protein was reduced by the attractive interactions between water and protein molecules. However, the difference in $G_{21}^{\alpha }$ between the helix dimer and coils, $\rho_{3}\left(G_{21}^{c}-G_{21}^{h}\right)$, namely, the water term with respect to $\Delta \Gamma_{23}$, would be mainly interpreted as the difference in the excluded volume between the helices and coils, namely $-\rho_{3}\left(V_{21}^{c}-V_{21}^{h}\right)$.

### 3.2 Solvation structures of TFE for the helix dimer and coil

As mentioned above, by utilizing the decomposition of $\Delta \Gamma_{23}$, the cosolvent and water term to $\Delta \Gamma_{23}$, namely, the direct contributions of cosolvent and water molecules to the structural stability of coiled‐coil helices of GCN4‐p1 in the aqueous solutions of TFE and urea were revealed. However, the molecular picture of the preferential solvation of cosolvent molecule toward the helix dimer and coils remained unclear. It has been suggested that HFIP and TFE have a high tendency to form micelle‐like assemblies in the bulk solutions and this ability maximizes at ~30 (v/v)% (Hirota et al., 1997; Hong et al., 1999). Such assemblies of alcohol molecules can potentially provide proteins/peptides a highly hydrophobic local environment in the same manner as micelles. Therefore, it has been suggested that the high ability of helix formation is induced upon binding of proteins to the hydrophobic clusters formed by alcohol molecules as that observed upon their binding to micelles (Hirota et al., 1997; Hong et al., 1999). However, at the low concentrations, where aqueous TFE bulk solution has no clustering formation but a helix induction ability, little has been characterized till date about the solvation structures of alcohol molecules around proteins.

**FIGURE 3:**
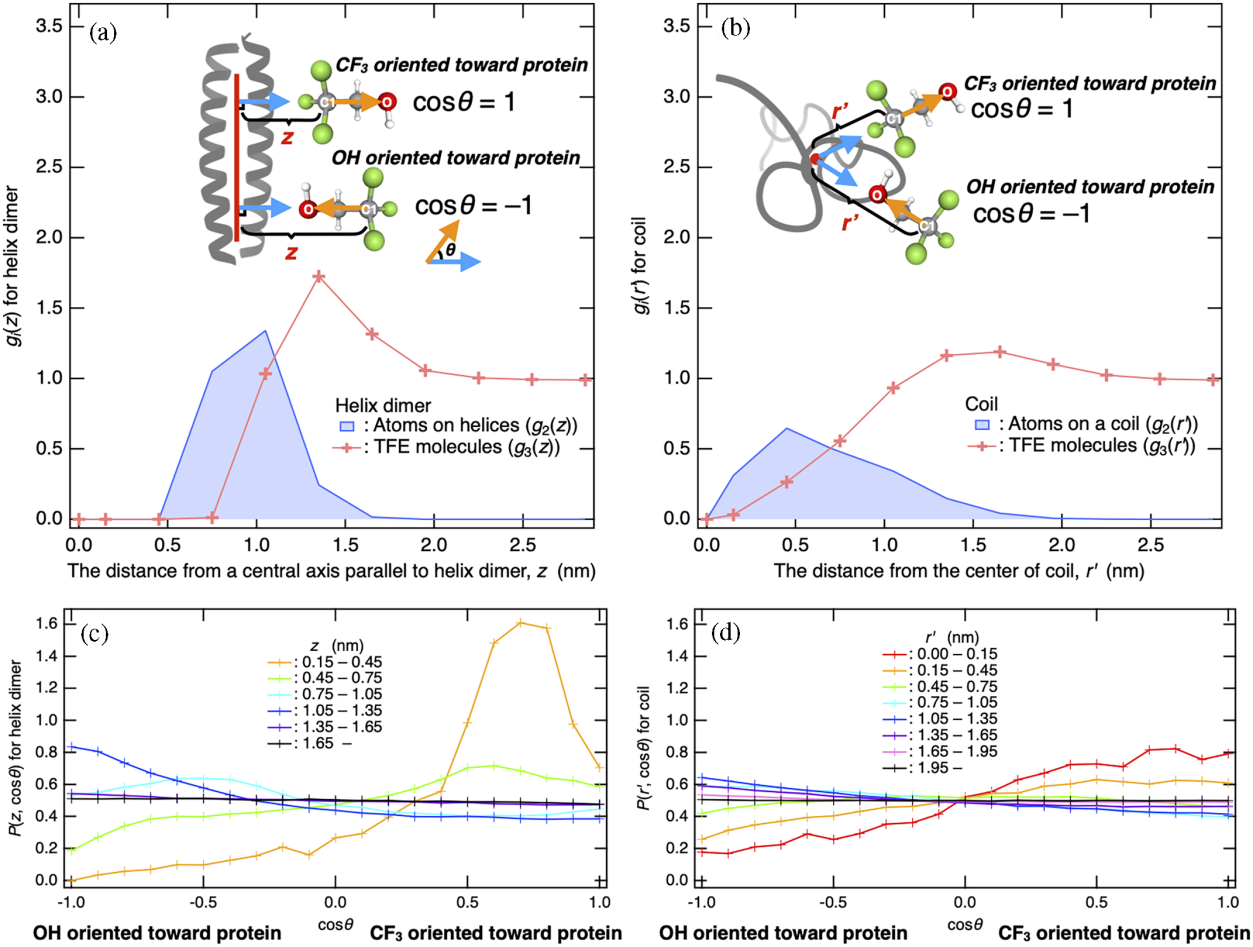
The formation of reverse‐micelle‐like orientations reinforces the solvation of TFE molecules to the helix dimer. (a) Distribution function of all atoms on the helix dimer $g_{2}\left(z\right)$ and that of TFE molecules $g_{3}\left(z\right)$ shown as a function of distance z from the central axis of the helix dimer. (b) Radial distribution function of the atoms on the coil $g_{2}\left(r^{\prime }\right)$ and that of TFE molecules $g_{3}\left(r^{\prime }\right)$ shown as a function of distance $r^{\prime }$ from the center of the coil obtained from the average positions of the atoms. (c) Distribution functions of the orientation of TFE molecule cosθ for each distance z between the central axis of the helix dimer and C1 atom of CF_3_ on TFE molecule, $P\left(z,\cos\;\theta \right)$. (d) Distribution functions of the orientation of TFE molecules cosθ for each radial distance $r^{\prime }$ between the center of the coil and C1 atom of TFE molecule, $P\left(r^{\prime },\cos\;\theta \right)$. cosθ=1 indicates that CF_3_ of TFE molecule points toward the protein, while cosθ=−1 indicates that the OH group of the TFE molecule points toward the protein. In the insets of (a), cosθ is defined by the inner product between a unit vector directed toward the C1 atom of TFE molecule (cyan arrows), which is vertical to the central axis of the helix dimer (colored red), and a unit vector that points toward the oxygen atom from the C1 atom of the TFE molecule (orange arrows). In the insets of (b), cosθ is defined by the inner product between a unit vector directed toward the C1 atom of TFE molecule from the center of atoms on the isolated coil (cyan arrows) and a unit vector that points toward the oxygen atom from the C1 atom of the TFE molecule (orange arrows). TFE, 2,2,2‐trifluoroethanol.

A distance distribution function of all the atoms of the helix dimer $g_{2}\left(z\right)$ and that of the carbon atom “C1” of CF_3_ in TFE molecules $g_{3}\left(z\right)$ as a function of the distance z from the central axis of the helix dimer are shown in Figure 3a. At the distances where the side chains of the helices are exposed to the solution (~0.75 < *z* < ~1.65 nm), $g_{3}\left(z\right)$ became large, indicating that TFE molecules form a solvation shell. For comparison, radial distribution functions of atoms on the coil, $g_{2}\left(r^{\prime }\right)$, and that of the C1 atom on TFE molecule, $g_{3}\left(r^{\prime }\right)$, are shown in Figure 3b as a function of the radial distance $r^{\prime }$ from the center of the coil obtained as the average of the coordinates of all atoms. The surfaces of the coil exposed to the solution were extended over the distances up to ~2 nm from ~0.75 nm, and thus $g_{3}\left(r^{\prime }\right)>1$ at these distances, while the maximum in $g_{3}\left(r^{\prime }\right)$ for the coil was smaller than that in $g_{3}\left(z\right)$ for the helix dimer, indicating the solvation of TFE molecules toward the coil was weaker.

To discuss the orientation of TFE molecule, we define cosθ as the inner product between a unit vector directing toward the C1 atom of TFE molecule, which is vertical to the central axis of the helix dimer, and a unit vector pointing toward the oxygen atom from the C1 atom of the TFE molecule (Figure 3a). Thus, cosθ=1 indicates that CF_3_ of the TFE molecule points toward the protein, while cosθ=−1 indicates that the OH group of the TFE molecule points toward the protein (Figure 3a). Distribution functions of the orientation of TFE molecule cosθ at given distances z between the central axis of the helix dimer and C1 atom of the TFE molecule, $P\left(z,\cos\;\theta \right)$, are shown in Figure 3c as a function of cosθ. For comparison, $P\left(r^{\prime },\cos\;\theta \right)$ for the coil was also analyzed; distribution functions of the orientation of TFE molecules cosθ for each radial distance $r^{\prime }$ between the center of the coil and C1 atom of the TFE molecule, $P\left(r^{\prime },\cos\;\theta \right)$, are shown in Figure 3d as a function of cosθ. The orientation of TFE molecule cosθ was defined by the inner product between a unit vector directing from the center of the coil toward the C1 atom of TFE molecule and the unit vector on TFE molecule introduced above (Figure 3a).

The CF_3_ groups of TFE molecules binding to the sites very close to the central axis of the helix dimer, at the distances of < ~0.75 nm, were observed to point toward the central axis (Figure 3c). Such TFE molecule was rarely populated ($g_{3}\left(z=0.75\;\right)=0.011),$ and thus its contribution to the PBP was small (Figure 3a). The binding sites of the CF_3_ group are expected to be located near the interfaces at which two helices interact via hydrophobic residues. In contrast, at the distances of ~0.75–~1.65 nm, where TFE molecules broadly populate and form a large solvation shell around the helix dimer, the OH groups of TFE molecules pointed toward the central axis of the helix dimer, indicating the formation of reverse‐micelle‐like orientations of TFE molecules. The similar reverse‐micelle‐like orientations of TFE were also found in a minimum‐distance distribution function (Martínez, 2022), where the hydroxy and trifluoromethyl groups of TFE were respectively located at the near and far sides from a peptide (Pereira et al., 2022). These results are consistent with previous MD simulations showing that peptides are not completely coated by TFE molecules and that the broad solvation shell contains a lot of water molecules to satisfy the solvation of exposed polar groups (Vymětal et al., 2016). This implies that in contrast to cases of reverse micelle formation, the OH groups of TFE molecules distributed around the protein can form hydrogen bonding with water molecules as well as polar groups of the protein.

Similar solvation structures were observed around the coil. The CF_3_ group of TFE molecules binding close to the center of the coil located at the distances $r^{\prime }$ less than ~0.75 nm pointed toward the center of the coil (Figure 3d). In contrast, at the distance larger than ~0.75 nm, where TFE molecules formed the solvation shell around the coil (Figure 3b), the OH groups of TFE molecules pointed toward the center of the coil (Figure 3d), indicating the formation of reverse‐micelle‐like orientations of TFE molecules, as was also seen for the helix dimer. Thus, in both the cases, the formation of reverse‐micelle‐like orientations of TFE molecules is expected to be favorable via electrostatic interactions between amino acid residues and OH groups of TFE molecules. On the other hand, the formation of reverse‐micelle‐like orientations does not result in the loss of hydrogen bonding of TFE molecules with surrounding water molecules (Vymětal et al., 2016), as mentioned above. As for the differences, because the reverse‐micelle‐like orientation of TFE molecules to the helix dimer is stronger than that to the coils (Figure 3c,d), TFE molecules were observed to form the larger solvation shells around the helix dimer than the coil (Figure 3a,b). The following section attributes this to the electrostatic interactions between the side chains of the helix dimer and OH groups of TFE molecules.

### 3.3 Decomposition analysis of interaction energies between protein and cosolvent

**FIGURE 4:**
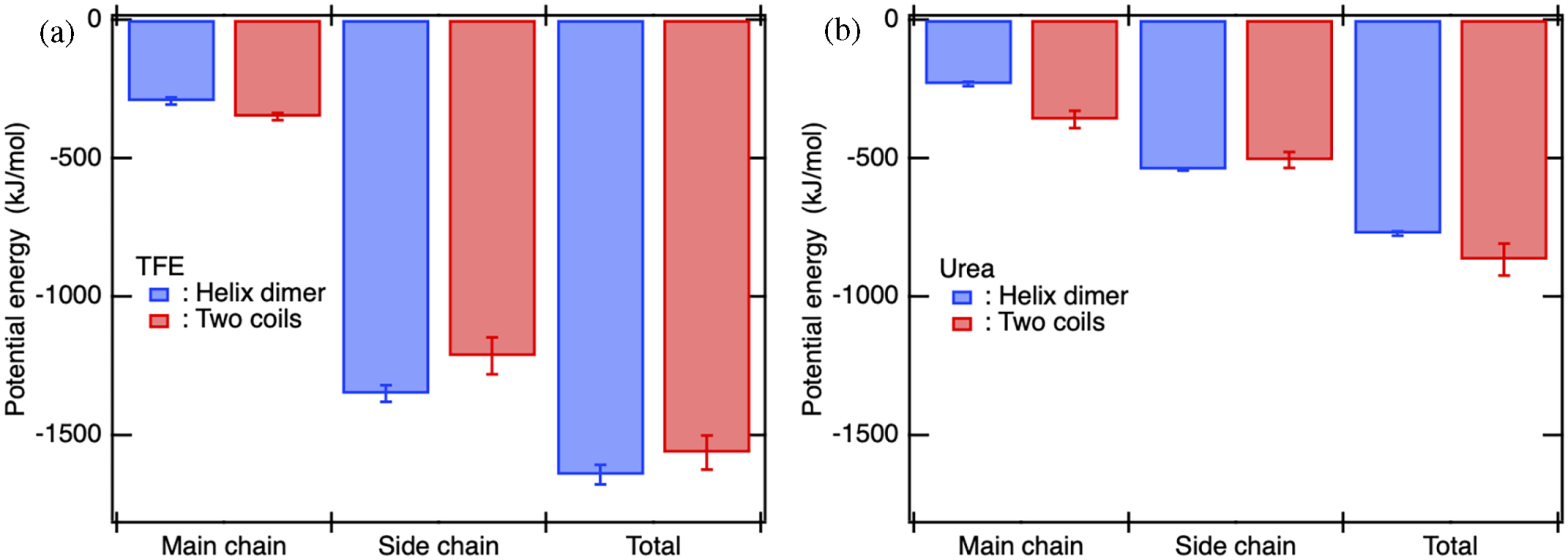
The large contribution from the cosolvent term in $\Delta \Gamma_{23}$ of TFE molecules toward the helix dimer and of urea molecules toward the coils can be respectively attributed to the direct interactions between the side chains of helices and TFE molecules and between the main chains of coils and urea molecules. (a) Decomposition analysis of the interaction energies between the helix dimer/coils and TFE molecules into the main chain and side chain parts. (b) A similar decomposition analysis has been shown for urea molecule. TFE, 2,2,2‐trifluoroethanol.

The direct interaction energy between the helix dimer/coils and TFE molecules and its decomposition into the main chain and side chain parts are shown in Figure 4a. The stronger solvation of TFE molecules toward the helix dimer than the coils (TFE term in Figure 1e) stemmed from the stronger direct interactions of TFE molecules with the helix dimer than the coils (Figure 4a). To be more specific, regardless of the conformation, the TFE molecules were found to interact more strongly with the side chains than with the main chains, and the total TFE‐protein direct interaction was dominated by its interactions with the side chains (Figure 4a). The stronger direct interactions of TFE molecules with the helix dimer than the coils could be mainly attributed to the interactions with the side chains rather the main chains of the helix dimer (Figure 4a).

A similar decomposition analysis for urea is shown in Figure 4b. The stronger solvation of urea molecules around the coils than the helix dimer (urea term in Figure 1f) stemmed from the stronger direct interactions of urea molecules with the coils than the helix dimer (Figure 4b). In both protein conformations, the absolute values of the total interaction energy with urea molecules were smaller than those with TFE molecules (Figure 4a,b). This was consistent with our observation that the radial distribution functions $g_{23}^{\alpha }\left(r\right)$ for urea were lower than that for TFE (Figure 1a,b), thereby, the KB integral $G_{23}^{\alpha }$ for urea were smaller than that for TFE (Figure 1c,d). Even though the interactions of the protein with urea molecules are not as strong as those with TFE molecules, the difference between the coils and helix dimer in terms of the interaction energies of urea molecules with the protein was sufficiently large and almost comparable with that of TFE molecules in the absolute value (Figure 4a); this was mainly due to the differences observed for the main chain parts (Figure 4b).

**FIGURE 5:**
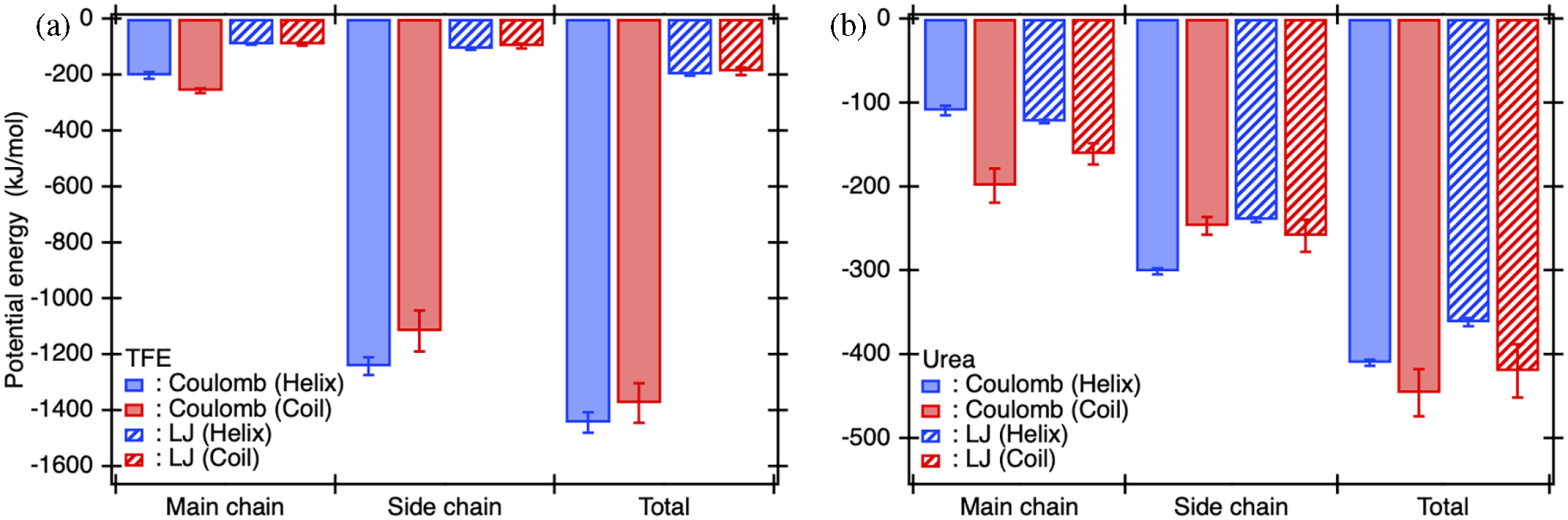
The difference between the helices and coils in terms of the total direct interaction energies of the protein with TFE/urea molecules can be respectively attributed to the electrostatic interactions between the side chains of helices and TFE molecules and to both the electrostatic and dispersion interactions between the main chains of coils and urea molecules. (a) Decomposition analysis of the total interaction energies and main chain and side chain parts between the protein and TFE molecules into the Coulomb interaction and Lennard‐Jones (LJ) interaction parts. (b) A similar decomposition analysis has been shown for urea molecules. TFE, 2,2,2‐trifluoroethanol.

From the decomposition analysis of interaction energies mentioned above, it was revealed that the parts of protein with which cosolvent molecules strongly interact depend on not only the kind of cosolvent but also the protein conformation. Next, to reveal the kind of interactions between the protein and TFE/urea molecules that result in the opposing cosolvent effects on protein stability, the total interaction energies and main chain and side chain parts between the protein and cosolvent molecules were further decomposed into the Coulomb and Lennard‐Jones (LJ) parts (Figure 5a for TFE and Figure 5b for urea). In the total interaction energies between the protein and TFE molecules, the electrostatic (Coulomb) interaction energies were dominant and yielded the difference observed between the helices and coils (Figure 5a). Furthermore, the difference in these electrostatic interaction energies was mainly attributed to the difference in the side chain parts (Figure 5a). In fact, TFE molecules formed larger extensive solvation shells around the helix dimer than those around the coils by directing the OH groups of TFE molecules toward the helix dimer (Figure 3c) and strengthening electrostatic interactions with the side chains of helix dimer. Therefore, the electrostatic interactions between the OH group of TFE molecules and the side chains of the helix dimer that were stronger than those observed for the coils (Figure 5a) resulted in the positive value of cosolvent term in the excess preferential solvation $\Delta \Gamma_{23}$ (Figure 1e). In a recent MD‐based study, it was demonstrated that inhibition of hydrogen bonds between the OH groups of TFE molecules and peptide backbone stabilizes α‐helices (Pereira et al., 2022). This inhibition should weaken the electrostatic interactions of TFE to the main chains of the coils more strongly than those to the helices, as expected from Figure 5a. This indicated that $\Gamma_{23}^{c}$ decreased more extensively than $\Gamma_{23}^{h}$, and thus $\Delta \Gamma_{23}$ increased, thereby resulting in the stabilization of α‐helices (Pereira et al., 2022).

By contrast, in the total interaction energies between the protein and urea molecules, the electrostatic and LJ interactions contributed almost comparably (Caflisch and Karplus, 1999; Canchi et al., 2010) and the differences in both these interaction energies yielded the difference in the total interaction energies between the helices and coils (Figure 5b). The side chain parts of both the electrostatic and LJ interaction energies contributed more to the total interaction energies than the main chain parts in both protein conformations (Figure 5b). However, the difference between the helices and coils in terms of the total electrostatic interaction energies and LJ interaction energies, respectively, could be predominantly attributed to the differences in the main chain parts (Berteotti et al., 2011; Lim et al., 2009) (Figure 5b). Therefore, the fact that both the electrostatic and dispersion (LJ) interactions of urea molecules with the main chains on the coils were stronger than these interactions with the main chains on the helix dimer resulted in the negative value of cosolvent term in the excess preferential solvation $\Delta \Gamma_{23}$ (Figure 1f).

### 3.4 The *m*‐values of GCN4‐p1 for helix formation by TFE and coil formation by urea

The information pertaining to cosolvent effects on the thermodynamic stability of proteins can be obtained from Equation (6) with the excess preferential solvation $\Delta \Gamma_{23}$. Using $\Delta \Gamma_{23}$ estimated by MD simulation at ~2 M TFE and urea, the *m*‐value for TFE and urea was calculated to be 0.15 and −0.95 kcal mol^−1^ M^−1^, respectively. Notably, the value of 0.15 for TFE is smaller than 1.43 for melittin (Hirota et al., 1997) and ~0.6 for D10 peptide with sequence DPAEAAKAGR (Jasanoff and Fersht, 1994). On the other hand, the *m*‐value for the alanine‐based peptide with sequence Ac‐AAAAAXAAAA‐NH_2_ was calculated by us to be 0.20 kcal mol^−1^ M^−1^ using α‐helical content determined by MD simulations at 2.7 M of TFE (Pereira et al., 2022). This value is comparable with the one that we estimated for GCN4‐p1. The current picture that the TFE term in $\Delta \Gamma_{23}$ depends on the electrostatic interaction between the side chains of protein molecules and TFE could be a reasonable explanation for the variation in *m*‐values for different sequences. The *m*‐value −0.95, which we determined for urea, is quantitatively consistent with the experimental value at pH 7, viz. −0.95 kcal mol^−1^ M^−1^ (Matousek et al., 2007).

### 3.5 Estimations of cosolvent concentration dependence of $\Delta \Gamma_{23}$ assuming the obtained *m*‐values

By assuming the *m*‐value estimated here, $\Delta \Gamma_{23}$ can be simulated as a function of [Co‐sol] (Figure S1). In the case of urea, $a_{33}$ provided by Equation (4) is nearly equal to 1 over a wide range of [urea] up to 8 M (Chitra and Smith, 2002); thus, no amplification of $\Delta \Gamma_{23}$ was induced by concentration fluctuations of aqueous urea solution (Figure S1b). Therefore, the mechanism that we have proposed for urea‐mediated stabilization of coils would be true even for high urea concentrations. In the case of TFE, $a_{33}$ decreased with increasing [TFE] and reached a minimum of ~0.16 at ~5 M TFE, and thus, $\Delta \Gamma_{23}$ was amplified by $a_{33}$. For instance, at 7 M of [TFE], $\Delta \Gamma_{23}$ for TFE was ~10, which is comparable with the absolute value of $\Delta \Gamma_{23}$ for urea (Figure S1a,b), even though the absolute *m*‐value for urea is more than six times larger than that for TFE. Such enhancement of helix‐induction coupled with the large concentration fluctuations of the cosolvent (>2 M) is unique for TFE, and has been discussed in our previous study (Ohgi et al., 2021). This could be predicted based on the molecular origin of TFE‐induced helix formation, that is, $\Delta \Gamma_{23}$, depicted at lower concentrations of TFE.

## 4 CONCLUDING REMARKS

In the present study, GCN4‐p1 with two‐stranded coiled coil helices was employed as a model protein to investigate the molecular origin of opposing cosolvent effects by TFE and urea on the thermodynamic stability of proteins. The two states, that is, the helix dimer and coils, were introduced to conduct the preferential binding analysis of TFE/urea molecules for these two states. In the helix dimer, the side chains are exposed to the aqueous mixture of cosolvents, while the main chains (peptide backbones) are buried inside the protein. By contrast, in the coils, the main chains as well as the side chains are exposed to the aqueous mixture of cosolvents. Such a simple difference in the conformation between the two states played a crucial role in terms of highlighting similarities and differences in the cosolvent effects by TFE and urea molecules as listed below.

1. Both TFE and urea molecules yielded positive values of PBP to the protein, $\Gamma_{23}^{\alpha }$, regardless of the protein conformation, whereas TFE and urea molecules respectively yielded positive and negative values for the excess preferential solvation $\Delta \Gamma_{23}$ (Figure 1c–f).
2. The cosolvent term, $\rho_{3}\left(G_{23}^{h}-G_{23}^{c}\right)$, was positive for TFE, indicating favorable condition for helix formation, while it was negative for urea, indicating favorable condition for coil induction (Figure 1e,f).
3. The water term, $\rho_{3}\left(G_{21}^{c}-G_{21}^{h}\right)$, was negative for TFE and urea solutions, thereby favoring coil induction (Figure 1e,f).
4. The negative water term observed in both TFE and urea solutions could be simply attributed to the larger excluded volume of the coils than the helix dimer, and this mechanism has been overlooked in urea‐induced stabilization of coils (Figure 2).
5. The positive cosolvent term by TFE molecules was caused by the stronger electrostatic interactions of the OH groups of TFE molecules with side chains of the helix dimer than those with the side chains of the coils (Figure 5).
6. The electrostatic interactions between the OH groups of TFE molecules and side chains of the helix dimer were found to be strengthened by the reverse‐micelle‐like orientation of TFE molecules around the helix dimer (Figure 3).
7. The negative cosolvent term by urea molecules stemmed from stronger electrostatic and dispersion interactions of urea molecules with the main chains of the coils than those with the main chains of the helix dimer (Figure 5).
8. Taken together, the molecular origin of the opposing cosolvent effects can be attributed to the chemical nature of these cosolvent molecules in that the OH groups of TFE molecule prefer to interact with polar side chains, whereas urea molecules prefer to interact with peptide backbones.

The analytical methods applied in this study may also be helpful in investigating the mechanisms of protein stabilization by sugars (Ajito et al., 2018b) and other substances (Ajito et al., 2018a).

### 4.1 Limitations of the study

The mechanism pertaining to TFE‐mediated stabilization of α‐helix demonstrated in this study has not been extensively validated for various proteins with a wide variety of sequences. However, the mechanism by the water term (water exclusion) favorable for disordered conformations must be qualitatively independent of the sequence and plays an important role commonly in urea‐induced denaturation of proteins. It is still unclear whether the mechanism that we proposed here for TFE‐mediated helix induction, especially, the formation of solvation shell with reverse‐micelle‐like orientation of TFE molecules, is true for high concentrations at which clustering of TFE molecules takes place. This study did not focus on the helical monomer, which is an alternative conformation observed upon addition of high concentration of TFE (Corrêa and Farah, 2007); this was because the main aim was to compare the effects of TFE and urea on the native and coiled states. Considering the larger surface area of the helical monomers than that of the helix dimer as well as their comparable excluded volume with the coil, both TFE and water terms are likely to stabilize the helical monomers more than the helix dimer. The new findings obtained here will provide insights into the understanding of the cosolvent effects of alcohols and urea on the thermodynamic stability of other proteins.

## 5 MATERIALS AND METHODS

### 5.1 MD simulations

MD simulations in the canonical (*NVT*) and isothermal–isobaric (*NPT*) ensembles were performed using the Gromacs 5.1.2 suite (Abraham et al., 2015). The Amber99SB force field (Hornak et al., 2006) for GCN4‐p1 and transferable intermolecular potential 3P (TIP3P) model (Jorgensen et al., 1983) for water were employed. A force field model for TFE parameterized using the generalized AMBER force field to reproduce experimental properties of pure liquid and water‐mixed solutions (Vymětal and Vondrášek, 2014) was employed for aqueous TFE solution. For aqueous urea solution, a force field model for urea parameterized to reproduce the experimental Kirkwood–Buff integrals (Weerasinghe and Smith, 2003) was employed. The GCN4‐p1 leucine zipper initial configuration for starting the MD simulations was obtained from its X‐ray structure (Protein Data Bank accession code: 2ZTA) (O'Shea et al., 1991). The amino acid residue charge states were set to pH 7, employing charge neutral states for histidine and asparagine. For the aqueous TFE solutions at the mole fraction of 0.04, a total of 15,310 and 7655 water molecules and 626 and 313 TFE molecules were added to the cubic box under periodic boundary conditions for the MD simulations of the two‐stranded coiled coil helices and coil monomer, respectively. In the same manner, for the aqueous urea solutions at the mole fraction of 0.04, a total of 14,100 and 7050 water molecules and 564 and 281 urea molecules were added for the MD simulations of the two‐stranded helices and the coil monomer, respectively. The length of one side of the simulation box was ~8.1 nm (TFE) and ~7.8 nm (Urea) for the two‐stranded coiled coil helices, and that was ~6.5 nm (TFE) and ~6.2 nm (Urea) for the coil monomer. Thus, the half of these lengths were the upper limit of the KB integrals in Equation (7). The KB integral for the coil monomer was well converged at 3.3 nm (Figure 1c) and 3.1 nm (Figure 1d). The plots of the KB values of the coil monomer at >3.3 nm (TFE, Figure 1c) and at >3.1 nm (Urea, Figure 1d) are extrapolated values, which are the same as the KB integral value at 3.3 nm and that at 3.1 nm, respectively. MD simulations of the two‐stranded helices and isolated coil monomer were performed in both the aqueous mixtures of cosolvents for 2 μs with 2.0 fs time step interval. Since monomeric helix underwent a spontaneous change into a coil, an initial conformation of coil monomer could easily be obtained from a short MD simulation of the monomer. The ensemble averages of thermodynamic quantities for the helix and coil state were calculated using 10^5^ configuration frames generated by the 2 μs‐MD simulations for the two‐stranded coiled coil helices and coil monomer, respectively. It is noted that one MD run is for sampling the helices and another MD run is for sampling the coil. This is because the two‐stranded helices and coil monomer keep their helical and coil conformations during the simulations, respectively. All standard deviations shown as error bars in this study were evaluated by decomposing the 10^5^‐conformation average into five equal block averages. The temperature of 300 K and pressure of 1 bar were controlled by Nosé–Hoover thermostat (Hoover, 1985; Nosé, 1984) and Parrinello–Rahman barostat (Parrinello and Rahman, 1981), respectively. Intramolecular bonds, including hydrogen atoms on GCN4‐p1, were constrained using the linear constraint solver for molecular simulations algorithm (Hess et al., 1997). Cutoff length of Lennard‐Jones and Coulomb potentials between interaction sites was set at 1.0 nm. Particle‐mesh Ewald method (Essmann et al., 1995) was used to take into consideration the long‐range correction for Coulomb interactions in all the MD simulations. On the other hand, a decomposition analysis of interaction energies between protein and cosolvent molecule was conducted using the Coulomb interaction energies calculated with the cutoff in the real space, that is, the electrostatic interactions with cosolvent molecules located near the protein surfaces. Gromacs “freevolume” was applied to calculate the excluded volume of the protein, wherein the approximate value was determined by particle insertion of water molecule with the radius of 0.14 nm. Notably, this “freevolume” method determines the occupied volume by the protein and would miss the protein's internal void (cavity) larger than water. While one cavity was detected for the helix dimer using McVol (Till and Ullmann, 2010), the contribution of the cavity volume to $V_{21}^{h}$ was small (~0.1%).

## CONFLICT OF INTEREST STATEMENT

The authors declare no conflicts of interest.

## Supporting information


**Data S1.** Supporting Information.

## References

[pro4763-bib-0001] Abraham MJ , Murtola T , Schulz R , Páll S , Smith JC , Hess B , et al. GROMACS: high performance molecular simulations through multi‐level parallelism from laptops to supercomputers. SoftwareX. 2015;1–2:19–25.

[pro4763-bib-0002] Ajito S , Hirai M , Iwase H , Shimizu N , Igarashi N , Ohta N . Protective action of trehalose and glucose on protein hydration shell clarified by using X‐ray and neutron scattering. Physica B: Condens Matter. 2018a;551:249–255.

[pro4763-bib-0003] Ajito S , Iwase H , Takata S‐I , Hirai M . Sugar‐mediated stabilization of protein against chemical or thermal denaturation. J Phys Chem B. 2018b;122:8685–8697. 30148630 10.1021/acs.jpcb.8b06572

[pro4763-bib-0004] Auton M , Holthauzen LMF , Bolen DW . Anatomy of energetic changes accompanying urea‐induced protein denaturation. Proc Natl Acad Sci. 2007;104:15317–15322. 17878304 10.1073/pnas.0706251104 PMC2000523

[pro4763-bib-0005] Bennion BJ , Daggett V . The molecular basis for the chemical denaturation of proteins by urea. PNAS. 2003;100:5142–5147. 12702764 10.1073/pnas.0930122100 PMC154312

[pro4763-bib-0006] Berteotti A , Barducci A , Parrinello M . Effect of urea on the β‐hairpin conformational ensemble and protein denaturation mechanism. J Am Chem Soc. 2011;133:17200–17206. 21854002 10.1021/ja202849a

[pro4763-bib-0007] Caballero‐Herrera A , Nordstrand K , Berndt KD , Nilsson L . Effect of urea on peptide conformation in water: molecular dynamics and experimental characterization. Biophys J. 2005;89:842–857. 15908578 10.1529/biophysj.105.061978 PMC1366634

[pro4763-bib-0008] Caflisch A , Karplus M . Structural details of urea binding to barnase: a molecular dynamics analysis. Structure. 1999;7:477–488. 10378267 10.1016/s0969-2126(99)80064-1

[pro4763-bib-0009] Canchi DR , García AE . Backbone and side‐chain contributions in protein denaturation by urea. Biophys J. 2011;100:1526–1533. 21402035 10.1016/j.bpj.2011.01.028 PMC3059734

[pro4763-bib-0010] Canchi DR , Paschek D , García AE . Equilibrium study of protein denaturation by urea. J Am Chem Soc. 2010;132:2338–2344. 20121105 10.1021/ja909348c

[pro4763-bib-0011] Chen X , Sagle LB , Cremer PS . Urea orientation at protein surfaces. J Am Chem Soc. 2007;129:15104–15105. 18001024 10.1021/ja075034m PMC2548331

[pro4763-bib-0012] Chitra R , Smith PE . Molecular association in solution: a Kirkwood‐Buff analysis of sodium chloride, ammonium sulfate, guanidinium chloride, urea, and 2,2,2‐trifluoroethanol in water. J Phys Chem B. 2002;106:1491–1500.

[pro4763-bib-0013] Corrêa F , Farah CS . Different effects of trifluoroethanol and glycerol on the stability of tropomyosin helices and the head‐to‐tail complex. Biophys J. 2007;92:2463–2475. 17218461 10.1529/biophysj.106.098541 PMC1864823

[pro4763-bib-0014] Das A , Mukhopadhyay C . Urea‐mediated protein denaturation: a consensus view. J Phys Chem B. 2009;113:12816–12824. 19708649 10.1021/jp906350s

[pro4763-bib-0015] de Oliveira IP , Martínez L . The shift in urea orientation at protein surfaces at low pH is compatible with a direct mechanism of protein denaturation. Phys Chem Chem Phys. 2019;22:354–367. 31815262 10.1039/c9cp05196a

[pro4763-bib-0016] Essmann U , Perera L , Berkowitz ML , Darden T , Lee H , Pedersen LG . A smooth particle mesh Ewald method. J Chem Phys. 1995;103:8577–8593.

[pro4763-bib-0017] Frank HS , Franks F . Structural approach to the solvent power of water for hydrocarbons; urea as a structure breaker. J Chem Phys. 1967;48:4746.

[pro4763-bib-0018] Ganguly P , Boserman P , van der Vegt NFA , Shea J‐E . Trimethylamine N‐oxide counteracts urea denaturation by inhibiting protein‐urea preferential interaction. J Am Chem Soc. 2018;140:483–492. 29214802 10.1021/jacs.7b11695

[pro4763-bib-0019] Gerig JT . Examination of trifluoroethanol interactions with Trp‐cage in trifluoroethanol‐water at 298 K through molecular dynamics simulations and intermolecular nuclear Overhauser effects. J Phys Chem B. 2019;123:3248–3258. 30916962 10.1021/acs.jpcb.9b01171

[pro4763-bib-0020] Hamada D , Kuroda Y , Tanaka T , Goto Y . High helical propensity of the peptide fragments derived from beta‐lactoglobulin, a predominantly beta‐sheet protein. J Mol Biol. 1995;254:737–746. 7500346 10.1006/jmbi.1995.0651

[pro4763-bib-0021] Hammes GG , Schimmel PR . An investigation of water‐urea and water‐urea‐polyethylene glycol interactions. J Am Chem Soc. 1967;89:442–446.

[pro4763-bib-0022] Herskovits TT , Gadegbeku B , Jaillet H . On the structural stability and solvent denaturation of proteins. I. Denaturation by the alcohols and glycols. J Biol Chem. 1970;245:2588–2598. 5445802

[pro4763-bib-0023] Hess B , Bekker H , Berendsen HJC , Fraaije JGEM . LINCS – a linear constraint solver for molecular simulations. J Comput Chem. 1997;18:1463–1472.

[pro4763-bib-0024] Hirota N , Mizuno K , Goto Y . Cooperative alpha‐helix formation of beta‐lactoglobulin and melittin induced by hexafluoroisopropanol. Protein Sci. 1997;6:416–421. 9041644 10.1002/pro.5560060218 PMC2143652

[pro4763-bib-0025] Hirota N , Mizuno K , Goto Y . Group additive contributions to the alcohol‐induced alpha‐helix formation of melittin: implication for the mechanism of the alcohol effects on proteins. J Mol Biol. 1998;275:365–378. 9466915 10.1006/jmbi.1997.1468

[pro4763-bib-0026] Hong DP , Hoshino M , Kuboi R , Goto Y . Clustering of fluorine‐substituted alcohols as a factor responsible for their marked effects on proteins and peptides. J Am Chem Soc. 1999;121:8427–8433.

[pro4763-bib-0027] Hoover WG . Canonical dynamics: equilibrium phase‐space distributions. Phys Rev A Gen Phys. 1985;31:1695–1697. 9895674 10.1103/physreva.31.1695

[pro4763-bib-0028] Hornak V , Abel R , Okur A , Strockbine B , Roitberg A , Simmerling C . Comparison of multiple Amber force fields and development of improved protein backbone parameters. Proteins. 2006;65:712–725. 16981200 10.1002/prot.21123 PMC4805110

[pro4763-bib-0029] Hua L , Zhou R , Thirumalai D , Berne BJ . Urea denaturation by stronger dispersion interactions with proteins than water implies a 2‐stage unfolding. PNAS. 2008;105:16928–16933. 18957546 10.1073/pnas.0808427105 PMC2579355

[pro4763-bib-0030] Ibarra‐Molero B , Zitzewitz JA , Matthews CR . Salt‐bridges can stabilize but do not accelerate the folding of the homodimeric coiled‐coil peptide GCN4‐p1. J Mol Biol. 2004;336:989–996. 15037063 10.1016/j.jmb.2003.12.069

[pro4763-bib-0031] Jasanoff A , Fersht AR . Quantitative‐determination of helical propensities from trifluoroethanol titration curves. Biochemistry. 1994;33:2129–2135. 8117669 10.1021/bi00174a020

[pro4763-bib-0032] Jorgensen WL , Chandrasekhar J , Madura JD , Impey RW , Klein ML . Comparison of simple potential functions for simulating liquid water. J Chem Phys. 1983;79:926–935.

[pro4763-bib-0033] Kauzmann W . Some factors in the interpretation of protein denaturation. Adv Protein Chem. 1959;14:1–63. 14404936 10.1016/s0065-3233(08)60608-7

[pro4763-bib-0034] Kentsis A , Sosnick TR . Trifluoroethanol promotes helix formation by destabilizing backbone exposure: desolvation rather than native hydrogen bonding defines the kinetic pathway of dimeric coiled coil folding. Biochemistry. 1998;37:14613–14622. 9772190 10.1021/bi981641y

[pro4763-bib-0035] Lim WK , Rösgen J , Englander SW . Urea, but not guanidinium, destabilizes proteins by forming hydrogen bonds to the peptide group. Proc Natl Acad Sci U S A. 2009;106:2595–2600. 19196963 10.1073/pnas.0812588106 PMC2650309

[pro4763-bib-0036] Liu YF , Bolen DW . The peptide backbone plays a dominant role in protein stabilization by naturally‐occurring osmolytes. Biochemistry. 1995;34:12884–12891. 7548045 10.1021/bi00039a051

[pro4763-bib-0037] Makhatadze GI , Privalov PL . Protein interactions with urea and guanidinium chloride – a calorimetric study. J Mol Biol. 1992;226:491–505. 1322462 10.1016/0022-2836(92)90963-k

[pro4763-bib-0038] Martínez L . ComplexMixtures.Jl: investigating the structure of solutions of complex‐shaped molecules from a solvent‐shell perspective. J Mol Liq. 2022;347:117945.

[pro4763-bib-0039] Matousek WM , Ciani B , Fitch CA , Garcia‐Moreno B , Kammerer RA , Alexandrescu AT . Electrostatic contributions to the stability of the GCN4 leucine zipper structure. J Mol Biol. 2007;374:206–219. 17920624 10.1016/j.jmb.2007.09.007 PMC2105789

[pro4763-bib-0040] Mirsky AE , Pauling L . On the structure of native, denatured, and coagulated proteins. Proc Natl Acad Sci U S A. 1936;22:439–447. 16577722 10.1073/pnas.22.7.439 PMC1076802

[pro4763-bib-0041] Nosé S . A molecular‐dynamics method for simulations in the canonical ensemble. Mol Phys. 1984;52:255–268.

[pro4763-bib-0042] Nozaki Y , Tanford C . The solubility of amino acids and related compounds in aqueous urea solutions. J Biol Chem. 1963;238:4074–4081. 14086747

[pro4763-bib-0043] Nozaki Y , Tanford C . The solubility of amino acids and two glycine peptides in aqueous ethanol and dioxane solutions. Establishment of a hydrophobicity scale. J Biol Chem. 1971;246:2211–2217. 5555568

[pro4763-bib-0044] Ohgi H , Imamura H , Sumi T , Nishikawa K , Koga Y , Westh P , et al. Two different regimes in alcohol‐induced coil‐helix transition: effects of 2,2,2‐trifluoroethanol on proteins being either independent of or enhanced by solvent structural fluctuations. Phys Chem Chem Phys. 2021;23:5760–5772. 33481971 10.1039/d0cp05103a

[pro4763-bib-0045] O'Shea EK , Klemm JD , Kim PS , Alber T . X‐ray structure of the GCN4 leucine zipper, a two‐stranded, parallel coiled coil. Science. 1991;254:539–544. 1948029 10.1126/science.1948029

[pro4763-bib-0046] O'Shea EK , Rutkowski R , Kim PS . Evidence that the leucine zipper is a coiled coil. Science. 1989;243:538–542. 2911757 10.1126/science.2911757

[pro4763-bib-0047] Parrinello M , Rahman A . Polymorphic transitions in single‐crystals – a new molecular‐dynamics method. J Appl Phys. 1981;52:7182–7190.

[pro4763-bib-0048] Pereira AF , Piccoli V , Martínez L . Trifluoroethanol direct interactions with protein backbones destabilize α‐helices. J Mol Liq. 2022;365:120209.

[pro4763-bib-0049] Pierce V , Kang M , Aburi M , Weerasinghe S , Smith PE . Recent applications of Kirkwood‐Buff theory to biological systems. Cell Biochem Biophys. 2008;50:1–22. 18043873 10.1007/s12013-007-9005-0 PMC2566781

[pro4763-bib-0050] Robinson DR , Jencks WP . The effect of compounds of the urea‐guanidinium class on the activity coefficient of acetyltetraglycine ethyl ester and related compounds. J Am Chem Soc. 1965;87:2461–2470. 10.1021/ja01089a028 14330716

[pro4763-bib-0051] Shibuta S , Imamura H . Hydration promoted by a methylene group: a volumetric study on alkynes in water. J Phys Chem B. 2018;122:2985–2991. 29486128 10.1021/acs.jpcb.8b00843

[pro4763-bib-0052] Shiraki K , Nishikawa K , Goto Y . Trifluoroethanol‐induced stabilization of the α‐helical structure of β‐lactoglobulin – implication for non‐hierarchical protein‐folding. J Mol Biol. 1995;245:180–194. 7799434 10.1006/jmbi.1994.0015

[pro4763-bib-0053] Smith PE . Chemical potential derivatives and preferential interaction parameters in biological systems from Kirkwood‐Buff theory. Biophys J. 2006;91:849–856. 16679363 10.1529/biophysj.105.078790 PMC1563761

[pro4763-bib-0054] Sonnichsen FD , Van Eyk JE , Hodges RS , Sykes BD . Effect of trifluoroethanol on protein secondary structure: an NMR and CD study using a synthetic actin peptide. Biochemistry. 1992;31:8790–8798. 1390666 10.1021/bi00152a015

[pro4763-bib-0055] Su Z , Dias CL . Molecular interactions accounting for protein denaturation by urea. J Mol Liq. 2017;228:168–175.

[pro4763-bib-0056] Sumi T , Imamura H . Water‐mediated interactions destabilize proteins. Protein Sci. 2021;30:2132–2143. 34382697 10.1002/pro.4168 PMC8442971

[pro4763-bib-0057] Tanford C . Protein denaturation. Adv Protein Chem. 1968;23:121–282. 4882248 10.1016/s0065-3233(08)60401-5

[pro4763-bib-0058] Tanford C . Protein denaturation. C. Theoretical models for the mechanism of denaturation. Adv Protein Chem. 1970;24:1–95. 4912353

[pro4763-bib-0059] Till MS , Ullmann GM . McVol – a program for calculating protein volumes and identifying cavities by a Monte Carlo algorithm. J Mol Model. 2010;16:419–429. 19626353 10.1007/s00894-009-0541-y

[pro4763-bib-0060] Uversky VN , Narizhneva NV , Kirschstein SO , Winter S , Lober G . Conformational transitions provoked by organic solvents in beta‐lactoglobulin: can a molten globule like intermediate be induced by the decrease in dielectric constant? Fold Des. 1997;2:163–172. 9218954 10.1016/s1359-0278(97)00023-0

[pro4763-bib-0061] Vymětal J , Bednarova L , Vondrášek J . Effect of TFE on the helical content of AK17 and HAL‐1 peptides: theoretical insights into the mechanism of helix stabilization. J Phys Chem B. 2016;120:1048–1059. 26786280 10.1021/acs.jpcb.5b11228

[pro4763-bib-0062] Vymětal J , Vondrášek J . Parametrization of 2,2,2‐trifluoroethanol based on the generalized AMBER force field provides realistic agreement between experimental and calculated properties of pure liquid as well as water‐mixed solutions. J Phys Chem B. 2014;118:10390–10404. 25110944 10.1021/jp505861b

[pro4763-bib-0063] Watlaufer DB , Malik SK , Stoller ML , Coffin RL . Nonpolar group participation in the denaturation of proteins by urea and guanidinium salts. Model compound studies. J Am Chem Soc. 1964;86:508–514.

[pro4763-bib-0064] Weerasinghe S , Smith PE . A Kirkwood−Buff derived force field for mixtures of urea and water. J Phys Chem B. 2003;107:3891–3898.

[pro4763-bib-0065] Whitney PL , Tanford C . Solubility of amino acids in aqueous urea solutions and its implications for the denaturation of proteins by urea. J Biol Chem. 1962;237:PC1735–PC1737. 14006647

[pro4763-bib-0066] Yang JJ , Buck M , Pitkeathly M , Kotik M , Haynie DT , Dobson CM , et al. Conformational properties of four peptides spanning the sequence of hen lysozyme. J Mol Biol. 1995;252:483–491. 7563067 10.1006/jmbi.1995.0513

[pro4763-bib-0067] Yang Z , Xiu P , Shi B , Hua L , Zhou R . Coherent microscopic picture for urea‐induced denaturation of proteins. J Phys Chem B. 2012;116:8856–8862. 22780326 10.1021/jp304114h

