# Supplementary material for: Molecular mechanism of the common and opposing cosolvent effects of fluorinated alcohol and urea on a coiled coil protein
Source: Protein Sci. 2023 Oct 1;32(10):e4763. doi: 10.1002/pro.4763 (PMC10519159; doi:10.1002/pro.4763)
Supplement: Supplementary file 1 — Data S1. Supporting Information. [file PRO-32-e4763-s001.pdf]

Molecular mechanism of the common and opposing co-solvent effects of fluorinated alcohol and urea on a coiled coil protein

Noa Nakata[1], Ryuichi Okamoto[2], Tomonari Sumi[1,3], Kenichiro Koga[1,3], Takeshi Morita[4], and Hiroshi Imamura[5]

[1]Department of Chemistry, Faculty of Science, Okayama University, 3-1-1 Tsushima-Naka, Kita-ku, Okayama 700-8530, Japan
[2]Graduate School of Information Science, University of Hyogo, 7-1-28 minatojima-Minamimachi, Chuo-ku, Kobe, Hyogo 650-0047, Japan
[3]Research Institute for Interdisciplinary Science, Okayama University, 3-1-1 Tsushima-Naka, Kita-ku, Okayama 700-8530, Japan
[4]Department of Chemistry, Graduate School of Science, Chiba University, Chiba 263-8522, Japan
[5]Department of Bio-Science, Nagahama Institute of Bio-Science and Technology, 1266 Tamura, Nagahama, Shiga 526-0829, Japan

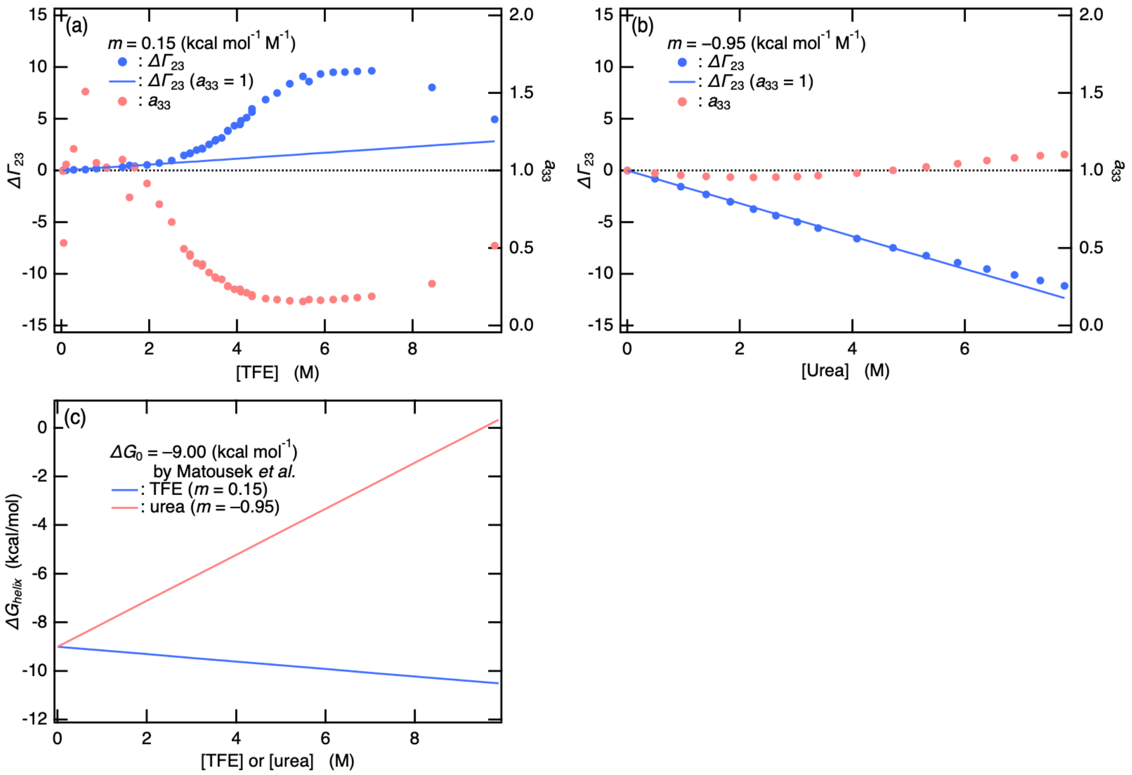

Figure S1. The excess preferential solvation to GCN4-p1 $\Delta\Gamma_{23}$ for TFE is amplified by a_{33} at TFE concentration higher than 2 M, while that for urea is not over the wide urea concentrations. (a) Aqueous TFE solution. (b) aqueous urea solution. (c) ΔG_{helix} for GCN4-p1 as a function of [Co-sol]. The experimental data for $\Delta G_0 = -9.0\ (\text{kcal mol}^{-1})$ is taken from literature [1]. The *m*-value, –0.95 (kcal mol^{-1} M^{-1}), which we determined for urea using the computational $\Delta\Gamma_{23}$ with Eq. 6, is consistent with the experimental data [1]. a_{33} is calculated using experimental KB integrals for aqueous TFE [2] and urea [3] solution.

Appendix

Relation between preferential binding and Gibbs energy for helix formation

The standard chemical potentials of the helix dimer ($\bar{\mu}_{\text{helix}}$) and two isolated coils ($\bar{\mu}_{\text{coil}}$) are related to the preferential binding parameter via the following equation [4]:

$$\beta\left(\frac{\partial\bar{\mu}_{\alpha}}{\partial\ln\rho_3}\right)_{T,P} = \beta\rho_3\left(\frac{\partial\bar{\mu}_{\alpha}}{\partial\rho_3}\right)_{T,P} = -\frac{\rho_3(G_{23}^{\alpha}-G_{21}^{\alpha})}{1+\rho_3(G_{33}-G_{31})} = -\Gamma_{23}^{\alpha}a_{33}, \qquad \text{(A1)}$$

where α is helix or coil, and Γ_{23}^{α} and a_{33} are provided by Eq. 1 and 4, respectively. If Γ_{23}^{α} is positive due to a preferential binding of cosolvent to protein, $\bar{\mu}_{\alpha}$ decreases with increasing ρ_3 by adding the cosolvent because of $a_{33} > 0$. If the Gibbs energy for helix formation $\Delta G_{\text{helix}} = \bar{\mu}_{\text{helix}} - \bar{\mu}_{\text{coil}}$ (Eq. 3b) becomes negative, i.e., $\bar{\mu}_{\text{helix}} < \bar{\mu}_{\text{coil}}$, with increasing ρ_3 by adding the cosolvent, Γ_{23}^{h} should be larger than Γ_{23}^{c}, namely, $\Delta\Gamma_{23} > 0$, therefore, the cosolvent more strongly solvates the helix dimer than the two isolated coil. In our previous study [2], we define ΔG_{helix} using two factors: the intramolecular interaction free energy of protein in a vacuum, E_{intra}, and solvation free energy of protein in aqueous solution, $\mu_{\text{s},2}$, as follows [5-7]:

$$\Delta G_{\text{helix}} = \bar{\mu}_{\text{helix}} - \bar{\mu}_{\text{coil}} = \left(E_{\text{intra}}^{\text{h}} + \mu_{\text{s},2}^{\text{h}}\right) - \left(E_{\text{intra}}^{\text{c}} + \mu_{\text{s},2}^{\text{c}}\right) \equiv \Delta E_{\text{intra}} + \Delta \mu_{\text{s},2}, \tag{A2a}$$

$$\Delta E_{\text{intra}} \equiv E_{\text{intra}}^{\text{h}} - E_{\text{intra}}^{\text{c}}, \tag{A2b}$$

$$\Delta \mu_{\text{s},2} \equiv \mu_{\text{s},2}^{\text{h}} - \mu_{\text{s},2}^{\text{c}}. \tag{A2c}$$

From Eq. A2, we obtain

$$\left(\frac{\partial \Delta G_{\text{helix}}}{\partial \rho_3}\right)_{T,P} = \left(\frac{\partial \Delta \mu_{\text{s},2}}{\partial \rho_3}\right)_{T,P}, \tag{A3}$$

because ΔE_{intra} does not depend on ρ_3, indicating that the cosolvent dependence of ΔG_{helix} is due to that of the difference in the solvation free energies $\Delta \mu_{\text{s},2}$. Using Eq. A3 and 3a, we obtain

$$\Delta \Gamma_{23} = -\frac{\beta \rho_3}{a_{33}} \left(\frac{\partial \Delta \mu_{\text{s},2}}{\partial \rho_3}\right)_{T,P}. \tag{A4}$$

Consequently, if the cosolvent more strongly solvates the helix dimer than the two isolated coils, namely, $\Delta \Gamma_{23}$, an addition of the cosolvent more largely decreases the solvation free energy of the helices than the coils.

References

1. Matousek WM, Ciani B, Fitch CA, Garcia-Moreno B, Kammerer RA, Alexandrescu AT (2007) Electrostatic contributions to the stability of the GCN4 leucine zipper structure. J. Mol. Biol. 374:206–219.

2. Ohgi H, Imamura H, Sumi T, Nishikawa K, Koga Y, Westh P, Morita T (2021) Two different regimes in alcohol-induced coil-helix transition: effects of 2,2,2-trifluoroethanol on proteins being either independent of or enhanced by solvent structural fluctuations. Physical Chemistry Chemical Physics 23:5760–5772.

3. Chitra R, Smith PE (2002) Molecular association in solution: A Kirkwood-Buff analysis of sodium chloride, ammonium sulfate, guanidinium chloride, urea, and 2,2,2-trifluoroethanol in water. J Phys Chem B 106:1491–1500.

4. Pierce V, Kang M, Aburi M, Weerasinghe S, Smith PE (2008) Recent applications of Kirkwood-Buff theory to biological systems. Cell Biochem Biophys 50:1–22.

5. Sumi T, Maruyama Y, Mitsutake A, Mochizuki K, Koga K (2018) Application of reference-modified density functional theory: Temperature and pressure dependences of solvation free energy. J. Comput. Chem. 39:202–217.

6. Sumi T, Koga K (2019) Theoretical analysis on thermodynamic stability of chignolin. Sci. Rep. 9:5186.

7. Sumi T, Imamura H (2021) Water-mediated interactions destabilize proteins. Protein Sci. 30:2132–2143.
